# Effect of Heat Treatment on Microstructures and Mechanical Properties of a Novel β-Solidifying TiAl Alloy

**DOI:** 10.3390/ma12101672

**Published:** 2019-05-23

**Authors:** Ning Cui, Qianqian Wu, Kexiao Bi, Tiewei Xu, Fantao Kong

**Affiliations:** 1 School of Mechanical and Automotive Engineering, Qingdao University of Technology, Qingdao 266520, China; wuqq2012@126.com (Q.W.); kexiaobiqd@sina.com (K.B.); twxu@163.com (T.X.); 2 State Key Laboratory of Advanced Welding and Joining, Harbin Institute of Technology, Harbin 150001, China; kft@hit.edu.cn

**Keywords:** TiAl alloy, β phase, heat treatment, mechanical properties

## Abstract

The effect of heat treatment on the microstructures and mechanical properties of a novel β-solidifying Ti–43Al–2Cr–2Mn–0.2Y alloy was investigated. A fully lamellar (FL) microstructure with a colony size of about 100 μm was obtained by heat treatment at 1320 °C/10 min/furnace cooling (FC). A duplex (DP) microstructure with globular γ grains and γ/α_2_ lamellae was obtained by heat treatment at 1250 °C/4 h/FC. The residual hard–brittle β_0_ phase was also eliminated after heat treatment. The mechanical properties of the β-solidifying TiAl alloy depended closely on the heat treatment. The FL alloy had better fracture toughness, and the fracture toughness (K_IC_) value was 24.15 MPa·m^1/2^. The DP alloy exhibited better ductility, and the room temperature (RT) elongation of the alloy could reach 1%. The elongation of the alloy with different microstructures sharply increased when the temperature increased from 700 to 750 °C, indicating that the microstructure had no effect on the ductile–brittle transition temperature of the β-solidifying TiAl alloy. The fracture morphologies of different tensile specimens were observed. Interlamellar and translamellar fractures were the main fracture features of the FL alloy. Intergranular, translamellar, and interlamellar fractures were the main fracture features of the DP alloy.

## 1. Introduction

γ-TiAl alloys are promising light-weight structural materials for high temperature application in aerospace industries owing to their high specific yield strength, high specific stiffness and good oxidation resistance [1,2,3]. However, there are still some problems involving low room temperature (RT) ductility and poor hot workability [4]. Microstructural refinement and homogenization are beneficial to the improvement of these properties [5]. A coarse as-cast microstructure can be transformed into fine grains by thermomechanical treatments [6]. Thus, novel β-solidifying TiAl alloys with excellent hot workability have attracted special attention recently. The disordered β phase is an elevated temperature ductile phase and can be introduced into TiAl alloys by adding β stabilizers [7]. A certain amount of β phase can remarkably enhance the hot deformability of TiAl alloys.

The mechanical properties of TiAl alloys depend closely on their microstructure. In order to further obtain desired mechanical properties, heat treatment is very essential for deformed TiAl alloys. Near gamma (NG), duplex (DP), nearly lamellar (NL), and fully lamellar (FL) are four typical microstructures of TiAl alloys. It is generally considered that FL and DP microstructures are suitable for engineering applications [5]. Previous studies on heat treatment have mainly focused on conventional (γ + α_2_) TiAl alloys and Nb-containing TiAl alloys. The microstructural control of these alloys is generally achieved through a multistep heat treatment at a very high temperature for a long time [8,9,10]. The complex heat treatment is related to the high microstructural stability and the slow diffusion of the alloying elements Nb and Mo [11,12]. Compared with conventional TiAl alloys and Nb-containing TiAl alloys, β-solidifying alloys contain more β stabilizers and phases, which affect the phase transition temperature and the position of phase fields [13]. The heat treatment of a β-solidifying TiAl alloy must have some new characteristics. However, limited research has been conducted on the heat treatment of β-solidifying TiAl alloys. Cr and Mn are typical β stabilizers and can significantly improve the ductility of TiAl alloys [14,15]. Up to now, the heat treatment of the Cr- and Mn-containing TiAl alloys has not been reported.

In this paper, the effect of heat treatment on a newly designed β-solidifying alloy (Ti–43Al–2Cr–2Mn–0.2Y) was investigated. The microstructural evolution, mechanical properties and fracture mechanisms of the alloy in different heat-treatment conditions were systematically studied.

## 2. Experimental Details

An as-cast ingot of the Ti–43Al–2Cr–2Mn–0.2Y (in at %) alloy was fabricated by induction skull melting and was then hot isostatically pressed (HIPed) at 1250 °C and 170 MPa for 4 h. An as-forged pancake was obtained through one-step canned forging with a reduction of 80%. The detailed description of the forging process has been reported in the literature [15]. All specimens for heat treatment were cut from the as-forged pancake by electrical discharge machining. Heat treatments were carried out in a 1600 °C heat-treatment furnace. Tensile tests and three-point bend tests were conducted using universal testing machines (Instron, Boston, Massachusetts, USA). The microstructure of the alloy in different conditions was examined by Quanta 200F scanning electron microscope (SEM, FEI, Hillsboro, OR, USA) in back-scattered electron mode (BSE). Transmission electron microscopy (TEM) observation was conducted on a Tecnai G2 F30 (FEI, Hillsboro, OR, USA). TEM specimens were prepared through mechanical polishing and twin-jet electropolishing by using a solution of 6 vol % perchloric acid + 34 vol % butanol + 60 vol % methanol at −20 °C and 25 V. 

## 3. Results and Discussion

### 3.1. Microstructure in the As-Cast and Forged Condition

**Figure 1:**
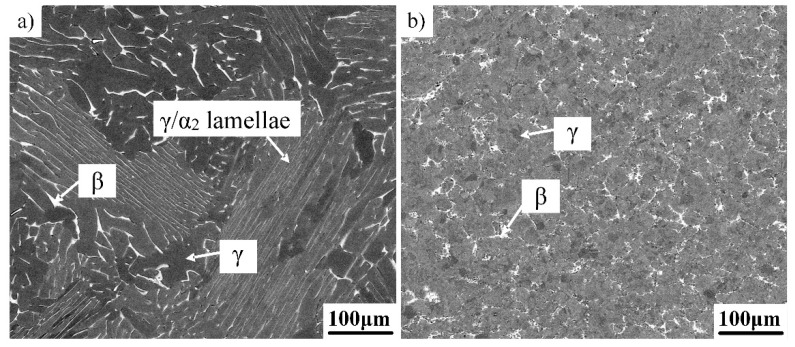
Initial microstructures of the Ti–43Al–2Cr–2Mn–0.2Y alloy. (**a**) As-cast, (**b**) as-forged.

Figure 1a shows the initial as-cast microstructure of the β-solidifying Ti–43Al–2Cr–2Mn–0.2Y ingot, which was mainly composed of coarse γ/α_2_ lamellar colonies and a small amount of γ and β_0_ phases situated at colony boundaries and triple junctions. It should be noted that disordered elevated temperature α and β phases are present as ordered α_2_ and β_0_ phases at RT, respectively. The coarse lamellar microstructure is detrimental to the mechanical properties of TiAl alloys. However, it is difficult to change the as-cast microstructure by heat treatment due to its high microstructure stability. In contrast, a deformed alloy is more suitable for heat treatment. Thus, the alloy was deformed through near-isothermal canned forging with a reduction of 80%. It can be seen from Figure 1b that the microstructure was significantly refined and homogenized after forging. The as-forged microstructure consisted of a fine equiaxed γ phase and a small amount of β_0_ phase. Nearly no α_2_ phase and residual lamellar colonies could be observed. The detailed investigation of the microstructure and phase constitution of the as-forged alloy has been reported in the literature [15]. Moreover, a fine as-forged microstructure can provide favorable conditions for subsequent heat treatment. Thus, the as-forged Ti–43Al–2Cr–2Mn–0.2Y alloy was chosen as the research object in this study.

### 3.2. Microstructure in Different Heat Treatment Conditions

A fine-grained FL microstructure is generally composed of only fine γ/α_2_ lamellar colonies, the formation of which depends closely on the phase transformation. The position of the phase boundaries in the Ti–Al phase diagram can be decreased by the addition of β stabilizers [13]. According to the Ti–Al phase diagram, the solidification path of a β-solidifying alloy can be described as follows: liquid → β → β + α (→α) → α + γ (+β) → α_2_ + γ (+β) [16,17]. In order to obtain an FL microstructure, the alloy should be heated to the single α phase field for a short time. After the microstructure is completely transformed into the α phase, the alloy should be cooled inside the furnace to RT. γ lamellae can be precipitated within the α phase during furnace cooling (FC) according to the Blackburn orientation relationship, thereby leading to the formation of γ/α_2_ lamellae. It should also be noted that the TiAl alloy would significantly deviate from thermodynamic equilibrium if a high cooling rate (air cooling or water cooling) was applied after a high-temperature heat treatment. FC is beneficial to obtain equilibrium γ/α_2_ lamellar microstructure and can effectively eliminate the residual stress. In order to obtain an optimum heat treatment process, actual experiments were conducted.

**Figure 2:**
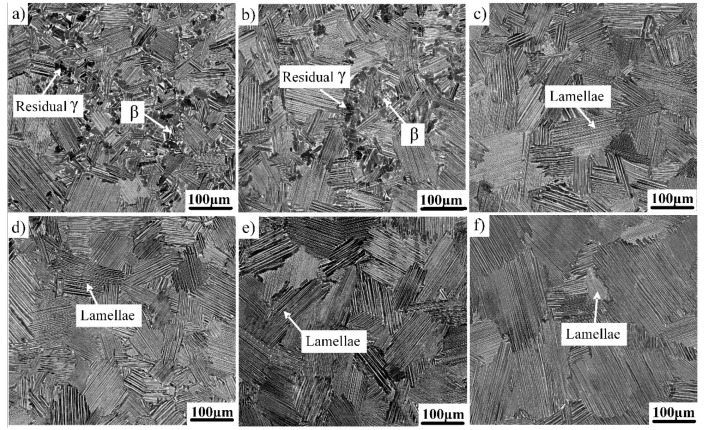
Lamellar microstructures of the as-forged Ti–43Al–2Cr–2Mn–0.2Y alloy in different heat treatment conditions: (**a**) 1300 °C/10 min, (**b**) 1310 °C/10 min, (**c**) 1320 °C/10 min, (**d**) 1340 °C/10 min, (**e**) 1360 °C/10 min, and (**f**) 1320 °C/2 h.

Figure 2 shows the microstructures of the as-forged Ti–43Al–2Cr–2Mn–0.2Y alloy after heat treatment at temperatures ranging from 1300 to 1360 °C followed by FC. The microstructure of the alloy after heat treatment at 1300 °C for 10 min (Figure 2a) showed that most initial as-forged microstructures were transformed into lamellar colonies. However, some residual globular γ and β phases still existed at colony boundaries and triple junctions, indicating that the alloy should be located in the (α + γ + β) phase field at 1300 °C. When the temperature increased to 1310 °C, the contents of residual γ and β phases significantly decreased (Figure 2b), indicating that the heat treatment temperature was still slightly lower. When the alloy was heat treated at 1320 °C for 10 min, the microstructure was almost completely composed of lamellar colonies with average colony sizes of about 100 μm, as shown in Figure 2c. This indicates that the alloy had entered into the single α phase field at 1320 °C. Zhang et al. reported that the α phase transition temperature (T_α_) of the Ti–43Al–9Nb–Y alloy was about 1400 °C, which is much higher than that of the present alloy. This is related to the diffusion ability of β-stabilizers. Slowly diffusing alloying elements such as Nb and Mo can increase the microstructural stability [11], while Cr and Mn exhibit high diffusion capacity and are beneficial to microstructural evolution [14,18,19]. The effect of higher temperatures on the lamellar colony size was also studied. As can be seen from Figure 2d,e, no abnormal grain growth was observed when the temperature further increased to 1340 °C and 1360 °C. The grain coarsening was inhibited due to the short holding time at the single α phase field. In order to further study the influence of extended holding time on the colony size, the alloy was heat treated at 1320 °C for 2 h. The corresponding microstructure is shown in Figure 2f. It can be seen that the colony size significantly increased. When the alloy was heated in the single α phase field, the growth of the α phase was not restricted, which can easily cause abnormal grain growth. Thus, it was necessary to control the holding time at the single α phase field. Based on the above analysis, the optimum heat treatment process of the FL microstructure should be 1320 °C/10 min/FC.

**Figure 3:**
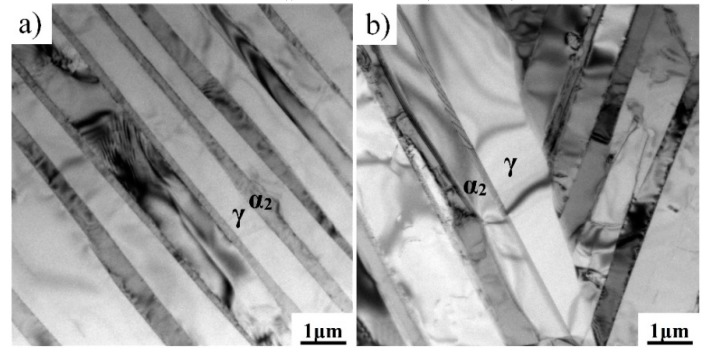
TEM images showing the microstructures of the as-forged Ti–43Al–2Cr–2Mn–0.2Y alloy after heat treatment at 1320 °C/10 min: (**a**) colony boundaries and (**b**) triple junctions.

As described above, the disordered elevated temperature β phase will be transformed into the hard and brittle β_0_ phase at RT. The nano hardness of the β_0_ phase is much higher than that of γ and α_2_ phases, which is detrimental to the compatible deformation among β_0_, γ, and α phases, thereby reducing the RT ductility of the TiAl alloy. Thus, it is desirable that the residual β_0_ phase in the as-forged microstructure can be sharply reduced or even eliminated by heat treatment. The elimination of the β_0_ phase is very difficult for the TiAl–Nb–Mo alloy, which is also related to the low diffusion capacity of Nb and Mo [20]. For the present alloy, Figure 2c shows that no β_0_ phase was observed in the SEM image. In order to further identify whether a small amount of β_0_ phase still existed at colony boundaries and triple junctions, TEM observations were conducted on the alloy after heat treatment at 1320 °C for 10 min, as shown in Figure 3. No β_0_ phase was observed at the colony boundaries and triple junctions in the FL microstructure, which suggests that the elevated temperature β phase had been completely transformed into γ and α_2_ phases during cooling.

A DP microstructure is generally composed of 50% globular γ grains and 50% γ/α_2_ lamellar colonies. According to the Ti–Al phase diagram, an equal amount of α and γ phases would form when the β-solidifying TiAl alloy is heated to the temperature in the middle of α + γ two-phase field [16]. Then, the γ phase would remain unchanged, and the α phase would transform into γ/α_2_ lamellae, thereby leading to the formation of a DP microstructure. Based on the above analysis, the TiAl alloy was heat treated at 1230–1270 °C for a long time to determine the optimum heat-treatment process of a DP microstructure.

**Figure 4:**
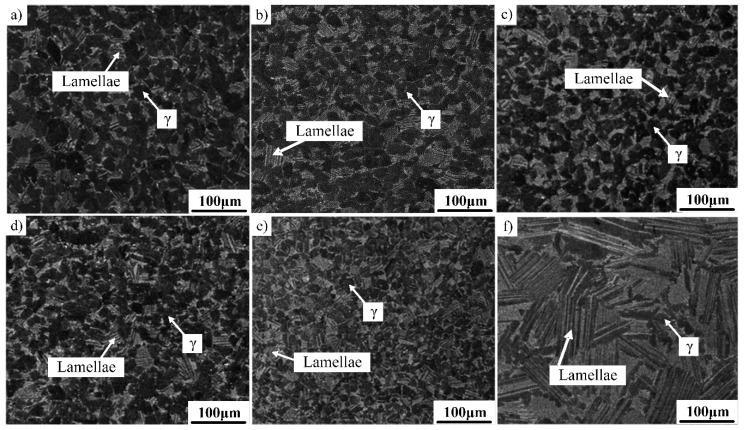
DP microstructure of the as-forged Ti–43Al–2Cr–2Mn–0.2Y alloy in different heat treatment conditions. (**a**) 1230 °C/4 h, (**b**) 1240 °C/4 h, (**c**) 1250 °C/4 h, (**d**) 1260 °C/4 h, (**e**) 1270 °C/4 h, (**f**) 1320 °C /10 min+1260 °C/4 h.

The microstructures of the as-forged Ti–43Al–2Cr–2Mn–0.2Y alloy in different heat-treatment conditions are shown in Figure 4. Figure 4a presents the microstructure of the alloy after heat treatment at 1230 °C for 4 h. It can be seen that the initial as-forged microstructure was transformed into a mixture of γ phase and γ/α_2_ lamellae. The content of the γ phase was higher than that of γ/α_2_ lamellae, indicating that 1230 °C should be located at the lower side of the γ + α phase field. Figure 4b shows the microstructure of the alloy after heat treatment at 1240 °C for 4 h. The γ/α_2_ lamellae content increased, while the γ phase content decreased. When the heat-treatment temperature increased to 1250–1260 °C, an equal amount of γ phase and γ/α_2_ lamellae could be obtained (Figure 4c,d). The average size of γ grains and lamellar colonies was about 10 and 20 μm, respectively, which is a typical DP microstructure. When the temperature further increased to 1270 °C, the γ/α_2_ lamellae content increased remarkably, as shown in Figure 4e. Moreover, according to the research on the Ti–43Al–6Nb–1B alloy, the FL microstructure can be obtained by the heat treatment of 1320 °C/10 min + 1260 °C/4 h [21]. The solidification path can be described as follows: α → α + γ → α_2_ + γ. The suitability of this heat treatment to the present alloy was also verified, as shown in Figure 4f. It can be observed that the deformed alloy was mainly composed of coarse lamellae and a small amount of γ phase. This indicates that the heat treatment is invalid for the present alloy. Thus, the optimum heat treatment process of DP microstructure should be 1250 °C/4 h/FC.

As shown in Figure 2c and Figure 4c, the grain size of the FL microstructure was much larger than that of the DP microstructure, which is related to the phase transformation during heat treatment. The growth of the α phase was not restricted by other phases when the alloy was heated at the single α phase field. In contrast, when the alloy was heat treated at the (α + γ) two-phase field, the growth of α and γ phases could be mutually restricted, thereby leading to the fine DP microstructure. Moreover, the heat treatment of the Cr- and Mn- containing TiAl alloy was simpler than that of conventional TiAl alloys and Nb-containing TiAl alloys. According to the Ti–Al phase diagram, T_α_ generally decreased with the decrease of the Al content. The Al content of the β-solidifying TiAl alloy was 42–43 at%, which was lower than that (47–48 at%) of the conventional TiAl alloy. Low Al content reduced T_α_. Furthermore, T_α_ can also be reduced by the addition of β–stabilizers [13]. Thus, the T_α_ of the β-solidifying TiAl alloy was lower than that of conventional TiAl alloys. For Nb-containing TiAl alloys, Nb has a high melting point and slow diffusion capacity, resulting in a high T_α_. The T_α_ of Ti-43Al-9Nb-Y is about 1400 °C, which is much higher than that of the present alloy.

### 3.3. Mechanical Properties

**Figure 5:**
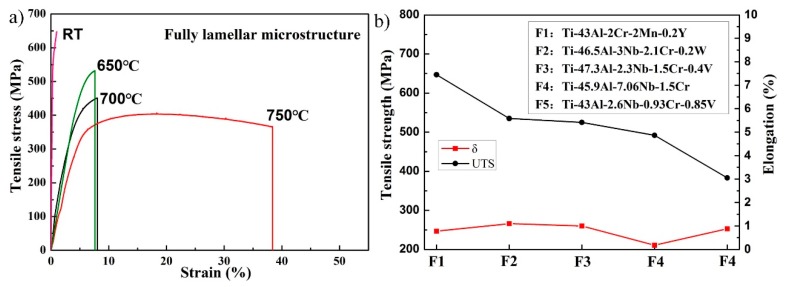
Tensile properties of as-forged TiAl alloys with FL microstructure: (**a**) tensile curves of the Ti–43Al–2Cr–2Mn–0.2Y alloy with FL microstructure, and (**b**) the comparison of tensile properties of various TiAl alloys with FL microstructure [24,25,26,27].

As-cast TiAl alloys generally exhibit poor mechanical properties due to their coarse initial microstructure. After forging, the ultimate tensile strength (UTS) and the elongation (δ) of the as-forged Ti–43Al–2Cr–2Mn–0.2Y alloy increased to 657 MPa and 0.86% at RT, respectively [15]. In order to study the effect of different microstructures on tensile properties, tensile tests were carried out at different temperatures between RT and 750 °C. Figure 5a shows the tensile properties of the Ti–43Al–2Cr–2Mn–0.2Y alloy with the FL microstructure. The UTS and δ of the FL alloy are 647 MPa and 0.78% at RT, respectively, which were slightly lower than that of the as-forged alloy. It is generally considered that an FL alloy exhibits higher strength than an as-forged alloy due to the resistance of lamellae on the dislocation glide [22,23], which is not consistent with the result of the current study. The microstructure of conventional as-forged TiAl alloys generally contains a large amount of residual γ/α_2_ lamellae, which is detrimental to the tensile property of TiAl alloys. The present as-forged TiAl alloy was mainly composed of fine equiaxed γ grains, which can enhance the UTS. When the temperature is 650 °C, the UTS and δ were 532 MPa and 1.8%, respectively. When the temperature increased to 700 °C, the UTS decreased to 451 MPa and δ increased to 7%. It can be found that δ dramatically increased to 32% when the temperature further increased to 750 °C, indicating that the ductile–brittle transition temperature (DBTT) of the FL alloy lies between 700 and 750 °C. Moreover, the RT mechanical properties of the present FL alloy were compared with that of other TiAl alloys, as shown in Figure 5b. It was found that δ of the present FL alloy was approximately that of other alloys, while the UTS of the present FL alloy was much higher. This indicates that the present FL alloy has better tensile properties, which should be benefited by the fine lamellar colony and the elimination of the brittle β_0_ phase.

**Figure 6:**
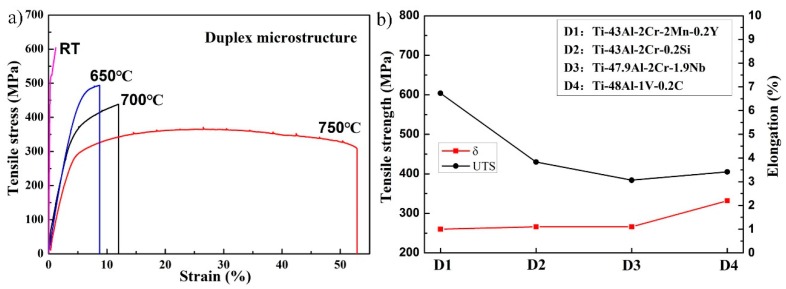
Tensile properties of as-forged TiAl alloys with DP microstructure: (**a**) tensile curves of the Ti–43Al–2Cr–2Mn–0.2Y alloy with DP microstructure, and (**b**) the comparison of tensile properties of various TiAl alloys with DP microstructure [28,29].

Figure 6a shows the tensile properties of the Ti–43Al–2Cr–2Mn–0.2Y alloy with the DP microstructure. The UST and δ were 604 MPa and 1% at RT, respectively. Compared with the as-forged alloy, the ductility of the DP alloy was significantly improved [22], which can be ascribed to the following reasons: (1) The DP microstructure was composed of fine γ grains and lamellar colonies, and the lamellae could block the crack propagation. (2) The dislocation density significantly decreased during the long heat treatment and work hardening was released. (3) The hard and brittle β_0_ phase was eliminated, which was beneficial to the compatible deformation among γ, α_2_, and β phases. When the temperature was 650 °C, the UTS and δ were 501 MPa and 3.4%, respectively. When the temperature increased to 700 °C, the UTS decreased to 438 MPa and δ increased to 7%, respectively. When the temperature further increased to 750 °C, δ sharply increased to 47%. The DBTT of the alloy with different microstructures was consistent with that of the as-forged alloy [15]. Figure 6b shows the comparison of tensile properties of various TiAl alloys with the DP microstructure. It can be found that the elongation of the present alloy was slightly lower than that of other alloys, while the present alloy exhibited higher tensile strength.

**Figure 7:**
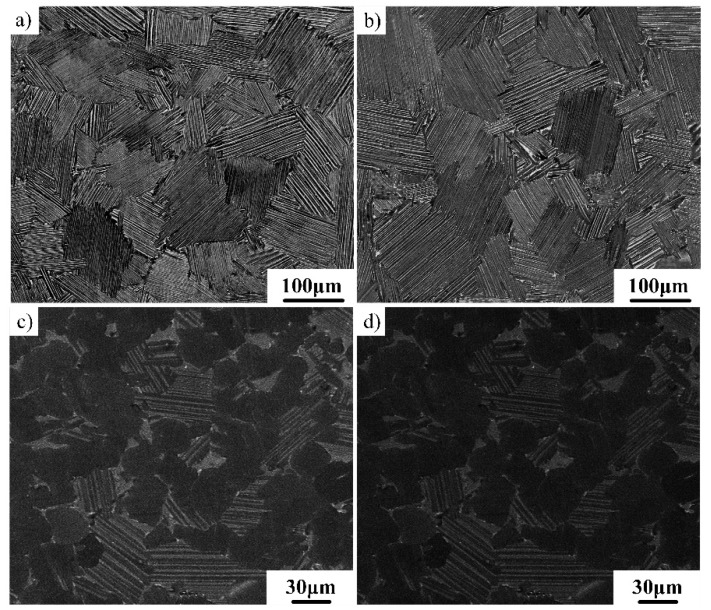
Quenched microstructure of TiAl alloys with different microstructures: (**a**) 700 °C/FL, (**b**) 750 °C/FL, (**c**) 700 °C/DP, and (**d**) 750 °C/DP.

**Figure 8:**
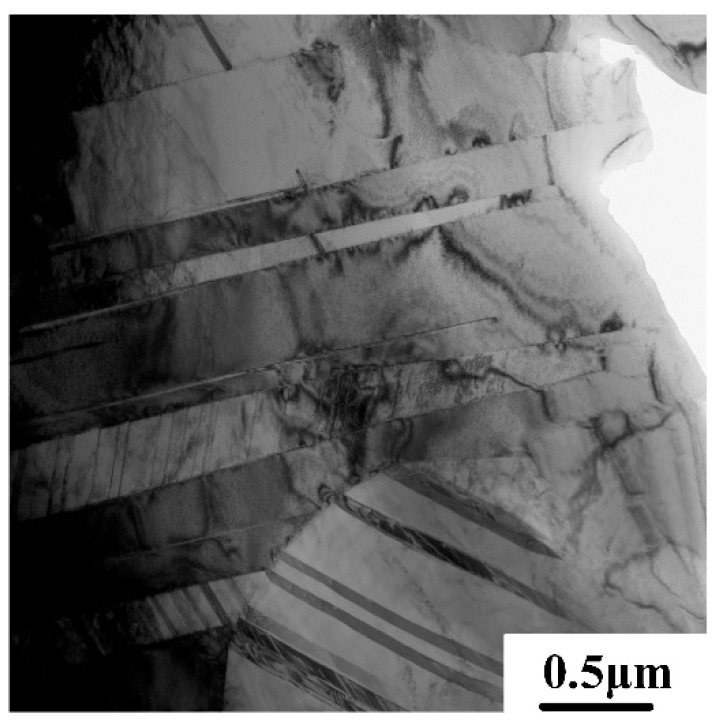
Mechanical twinning in a Ti–43Al–2Cr–2Mn–0.2Y alloy with FL microstructure observed after tensile deformation at 750 °C.

The relationship between the brittle-ductile transition and the microstructure was also studied. According to the heating conditions of tensile tests, both FL and DP alloys were heated to 700 and 750 °C for 2 min and then quenched to room temperature in water. The quenched microstructures are shown in Figure 7. It can be seen that there was no significant difference between the initial and quenched microstructures, which can be ascribed to the short holding time, high melting point, and high microstructure stability. Thus, the microstructural evolution should not be the main reason for the brittle–ductile transition. Previous studies have confirmed that the brittle–ductile transition of the TiAl alloy depends closely on the ductility of the γ and α_2_ phases. The deformation of TiAl alloys at 700–800 °C is mainly carried by the γ phase, which is similar to the deformation at room temperature [14]. The improved ductility of the γ phase has been attributed to the unpinning of the faulted partial dislocations with Burgers vector b, given by b = 1/6<112] [30,31], and more recently to the improved mobility of the dislocations with b = 1/2<110] at elevated temperatures [32]. Besides the dislocation glides, the climb of ordinary dislocations also contributes to the deformation at 700–800 °C [33]. Compared with room-temperature deformation, the activation of mechanical twinning within the γ phase was also enhanced. As shown in Figure 8, mechanical twinning can be found in the present alloy after tensile deformation at 750 °C. A similar phenomenon was also observed in the Ti–48Al–2Cr alloy after tensile deformation at 800 °C [34]. Moreover, the deformability of the α_2_ phase was enhanced when the temperature increased to 700–800 °C, which was benefited by a homogeneous activation of prismatic glide and a relatively dense population of 1/3<11$\bar{2}$6> dislocations on pyramidal planes [35]. Thus, the brittle-ductile transition of the TiAl alloy should be ascribed to the enhanced ductility of γ and α_2_ phases.

**Figure 9:**
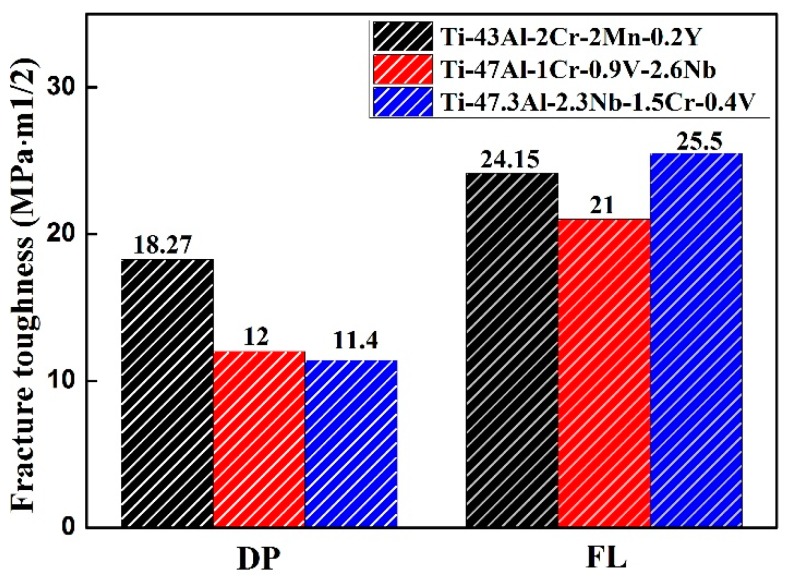
Fracture toughness of various TiAl alloys [25,36].

The fracture toughness of the as-forged Ti–43Al–2Cr–2Mn–0.2Y alloy in different conditions was also studied. The fracture toughness (K_IC_) value of the as-forged TiAl alloy was 14.63 MPa·m^1/2^. A low K_IC_ can be ascribed to the absence of γ/α_2_ lamellae. The as-forged TiAl alloy mainly consisted of equiaxed γ grains. Cracks occurred easily by cleavage through γ grains, resulting in low toughness. The K_IC_ values of TiAl alloys with different microstructures are shown in Figure 9. The Ti–43Al–2Cr–2Mn–0.2Y alloy with the fine-grained DP microstructure had a fracture toughness of 18.27 MPa·m^1/2^, which is higher than other TiAl alloys. In the present alloy, crack advance occurred by cleavage through γ grains, and could then be inhibited by boundaries of γ/α_2_ lamellae. In contrast, the lamellar colony in the FL alloy was larger than that in the DP alloy, which would provide larger resistance for the crack propagation. So, the Ti–43Al–2Cr–2Mn–0.2Y alloy with the FL microstructure exhibited higher fracture toughness. The K_IC_ value could reach 24.15 MPa·m^1/2^, which is close to that of the other alloy.

### 3.4. Fracture Mechanism

**Figure 10:**
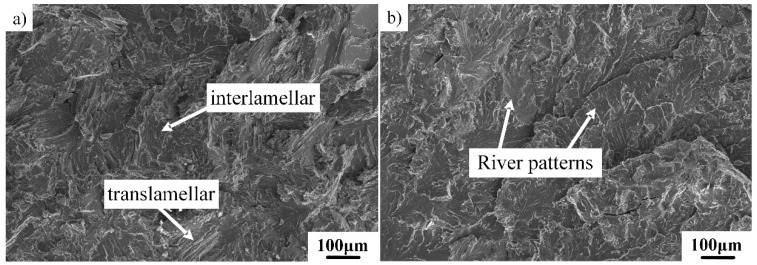
RT tensile fracture surfaces of Ti–43Al–2Cr–2Mn–0.2Y alloy with FL microstructure: (**a**) fracture feature and (**b**) river patterns.

The fracture behavior of the β-solidifying Ti–43Al–2Cr–2Mn–0.2Y alloy with various microstructures was investigated. Figure 10 shows the fracture morphology of the tensile specimen with the FL microstructure. As can be seen from Figure 10a, the interlamellar fracture was the main fracture feature for the FL alloy, while the translamellar fracture occurred occasionally. The lamellar colony generally contains many γ and α_2_ lathes. It is difficult to transfer across these γ/α_2_ interfaces through dislocation slip due to the different crystal structures of γ and α_2_ phases [37]. Thus, cracks tend to propagate along γ/α_2_ interfaces, leading to an increase in interlamellar fracture. The crack may pass through the γ/α_2_ lamellae only when the crack is nearly perpendicular to the lamellar interface. As shown in Figure 10b, river patterns, which are the typical feature of interlamellar fracture, could be clearly observed. These patterns suggest the local crack propagation direction.

**Figure 11:**
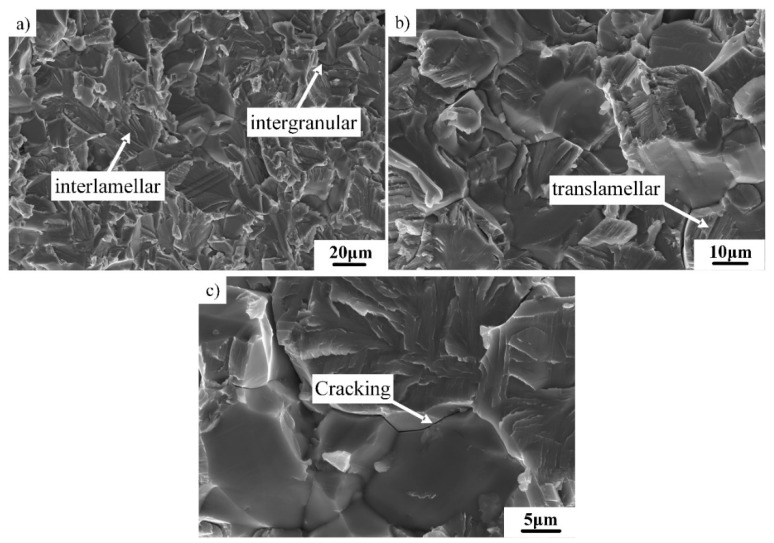
RT tensile fracture surfaces of Ti–43Al–2Cr–2Mn–0.2Y alloy with DP microstructure: (**a**,**b**) fracture feature and (**c**) cracking.

Figure 11 shows the fracture morphology of the tensile specimen with the DP microstructure. As shown in Figure 11a,b, three fracture features can be identified at RT: Intergranular, translamellar, and interlamellar fractures. Intergranular fracture is the main fracture mechanism in the γ grain accumulation region. The crack cannot pass through γ grains due to their small size and tends to propagate along grain boundaries of the γ phase. In contrast, translamellar and interlamellar fractures coexist simultaneously in the lamella accumulation region, which can be attributed to the small colony size. The propagation direction of the crack is correlated with the local stress and the crystal orientation of γ/α_2_ phases [37]. When the angle (η) between the crack and the lamellar interface is small, the crack tends to propagate along the lamellar interface. When η is about 90°, the crack may pass through γ/α_2_ lamellae. As shown in Figure 11c, cracking between two grains could clearly be identified, which further proved the occurrence of an intergranular fracture.

## 4. Conclusions

The effect of heat treatment on the microstructural evolution, mechanical properties, and fracture mechanisms of a novel β-solidifying Ti–43Al–2Cr–2Mn–0.2Y alloy was systematically investigated in this paper. The main conclusions from this work are as follows:

(1) An FL microstructure with a colony size of about 100 μm could be obtained through heat treatment at 1320 °C/10 min/FC. A DP microstructure composed of globular γ grains and fine γ/α_2_ lamellar colonies could be obtained after heat treatment at 1250 °C/4 h/FC. The hard and brittle β_0_ phase could be eliminated by the proper heat treatment.
(2) The alloy with the FL microstructure had better fracture toughness, and the K_IC_ was 24.15 MPa·m^1/2^. The RT elongation of the alloy with the DP microstructure could reach 1%. The elongation of the alloy with different microstructures dramatically increased as the temperature was raised from 700 to 750 °C, indicating that the microstructure had no effect on the ductile–brittle transition temperature of the β-solidifying TiAl alloy.
(3) Interlamellar and translamellar fractures were the main fracture features of the FL alloy. Intergranular, translamellar, and interlamellar fractures were the main fracture features of the DP alloy.
